# Free hemi-hamate arthroplasty: A review of donor site outcomes^✰^^✰✰^

**DOI:** 10.1016/j.jpra.2024.03.009

**Published:** 2024-03-28

**Authors:** Robert Phan, Yi Xie, Ishith Seth, Connor J. Atkinson, Damon Thomas, David J. Hunter-Smith, Warren M. Rozen, Roberto Cuomo

**Affiliations:** a Peninsula Health, Frankston Hospital, 2 Hastings Road, Frankston, Victoria 3199, Australia; b Department of Medicine, Surgery and Neuroscience, University of Siena, Italy

**Keywords:** Donor Site, Arthroplasty, Hamate, Closure, Proximal interphalangeal joints, PIPJ

## Abstract

**Introduction:**

The use of the dorsal hamate as a free osteochondral bone graft or vascularized bone flap has become the mainstay for large, comminuted middle phalanx volar lip fractures. To date, few studies have been conducted in the assessment of donor site morbidity for the hemi-hamate graft or flap, and none have discussed modes of repair or reconstruction of this donor site.

**Methods:**

A retrospective analysis of 14 hemi-hamate arthroplasty (HHA) procedures, including 6 vascularized and 8 non-vascularized grafts, from two surgeons was performed. Four hamate defect reconstruction techniques were utilized: no formal reconstruction, autologous bone grafting, gel foam, or synthetic bone substitute. The dorsal capsule was repaired with either extensor retinaculum grafting or by direct closure. Wrist range of motion, pain scores, and radiographic alignment were assessed.

**Results:**

At 6 months follow-up, all patients achieved full, pain-free wrist motion compared to the uninjured side, with visual analog scale pain scores of 0. Serial radiographs showed maintained carpal alignment without instability or subluxation. No differences based on the hamate defect reconstruction method or capsular repair technique was demonstrated.

**Conclusion:**

Safe return to pain free, unrestricted wrist function is achievable after HHA, regardless of hamate donor site management. Adequate dorsal capsular repair appears critical to prevent instability. Further study is needed to compare techniques, but choice may be guided by surgeon preference in the absence of clear evidence.

## Introduction

Comminuted fractures of the proximal interphalangeal (PIP) joint present a challenging problem in hand surgery. These injuries carry a high risk of persistent stiffness and deformity regardless of whether they are managed conservatively or with operative intervention.^1^ Various surgical techniques have been described,^2^ with the hemi-hamate arthroplasty (HHA) becoming a widely utilized option for comminuted, unstable PIP joint fractures. This technique, pioneered by Hastings et al. in 1999, involves the use of an osteochondral autograft harvested from the patient's distal hamate to reconstruct the volar aspect of the middle phalangeal base. The HHA involves harvesting an osteochondral graft from the hamate to reconstruct the PIP joint surface, with the aim of restoring joint congruity and stability, allowing early mobilization.^3^ The anatomical congruence between the dorsal hamate's articular surface and the volar lip of the middle phalanx base underpins the success of this procedure, allowing for the restoration of joint congruity and the facilitation of early mobilization.

While traditionally harvested as a non-vascularized osteochondral graft, issues with progressive graft loss and subsequent osteoarthritis at the PIP joint have been reported.^3^^,^^4^ Using a vascularized osteochondral flap including the hamate, wrist capsule, and overlying soft tissue has been postulated to improve graft survival.^5^^,^^6^ However, this creates a more significant defect at the HHA donor site. Despite the increasing adoption of HHA and its documented success in restoring PIPJ function, the procedure's implications on the donor site—the dorsal hamate—remain underexplored. The dorsal hamate plays a pivotal role in the hand's biomechanics, particularly in the mobility and stability of the 4th and 5th carpometacarpal joints (CMCJs), which are crucial for precision grips and power. The harvesting of a hemi-hamate osteochondral graft thus raises concerns regarding potential donor site morbidity, including alterations in the anatomy and biomechanics of the 4th and 5th CMCJs, as well as the overall impact on hand function.

Much of the focus in the literature has been on functional outcomes and complications related to the reconstructed PIP joint. Given the paucity of literature focusing on the consequences of hemi-hamate graft harvesting and donor site morbidity following HHA, especially with vascularized flaps. This case series reports on the assessment of wrist pain, range of motion, and carpal stability after HHA, along with analysis of capsular and bone reconstruction techniques.

## Materials and methods

We performed a retrospective review of all HHAs for comminuted PIP joint fractures undertaken by two specialist hand surgeons between January 2018 and July 2019. Institutional review board approval was obtained prior to commencing the study. Medical records, operative notes, and hand therapy reviews were analyzed to collect the following data: patient demographics, graft vascularity, donor site closure method, and postoperative donor site morbidity.

### Surgical technique

The hemi-hamate was harvested through a dorsal longitudinal incision overlying the fourth and fifth metacarpal bases. The skin was retracted, and blunt dissection preserved the extensor tendons and superficial nerve branches. For the free flap HHA, the transverse carpal arch was identified, passing within the supra-capsular plane. This was traced proximally to identify the main arterial and venous pedicles for microvascular anastomosis. The carpal arches were also followed distally, beyond the radial border of the hamate, to identify the continuation of the artery for consideration of use as a ‘flow-through’ flap. The dorsal surface of the hamate was then exposed, and the distal joint capsule was incised and reflected proximally to expose the CMCJ surface. Careful dissection was performed to preserve the periosteal attachments of the joint capsule. Osteochondral graft dimensions were then measured and marked distally on the hamate. The hemi-hamate segment was sharply excised using an oscillating saw and osteotomes. Four different techniques were utilized for reconstruction of the donor hamate defect: 1) No formal reconstruction of the bony defect; 2) Packed autologous cancellous bone graft obtained from the fractured PIP joint; 3) Insertion of gel foam into the defect; 4) Use of LMT BonAlive synthetic bone graft. The dorsal wrist capsule was then reconstructed with either a segment of extensor retinaculum graft, secured with 3–0 Tycon, or directly closed with 3–0 Vicryl. The overlying subcutaneous tissues and skin were closed in layers with 4–0 Monocryl. Revascularization of the free flap HHA was achieved by microsurgical anastomosis of each end of the transverse carpal arch to cut ends of the ulnar digital artery to the finger, ensuring no sacrifice to a digital vessel and ‘flow-through’ arterial supply to the flap.

### Postoperative management and follow-up

Postoperatively, patients were discharged home in a volar wrist splint with the wrist held in approximately 10–30° of extension. Patients were given instructions for strict wrist splinting for 4 weeks. Active mobilization of all finger joints commenced on the first operative day, using a 20° extension block splint at the PIP joint. After 3 weeks, the extension block was removed, and full range of motion (ROM) was permitted. After 4 weeks, the wrist splint was discontinued, and a gentle active wrist ROM was initiated under hand therapist guidance. Patients were followed up clinically and radiographically over 6 months. Outcomes assessed included wrist ROM compared to the uninjured side, and pain levels utilizing a visual analog scale (VAS). Plain radiographs were undertaken at each point of follow-up to assess carpal alignment and donor site healing. Donor site morbidity was defined as limited ROM compared with the uninjured side, ongoing pain at 6 months as recorded on a VAS, or evidence of CMCJ subluxation or dislocation at the fourth and fifth CMCJs as demonstrated on radiographs.

## Results

**Figure 1:**
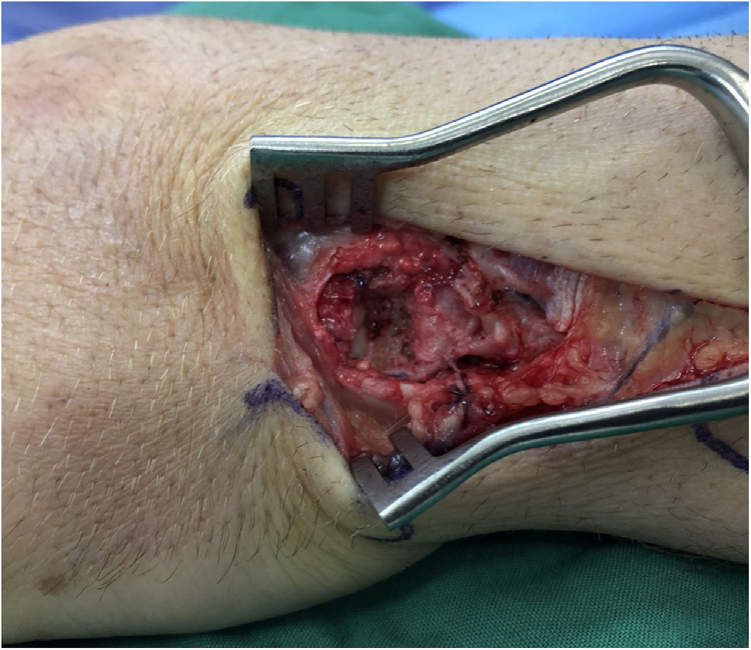
Hemi-Hamate donor site defect.

**Figure 2:**
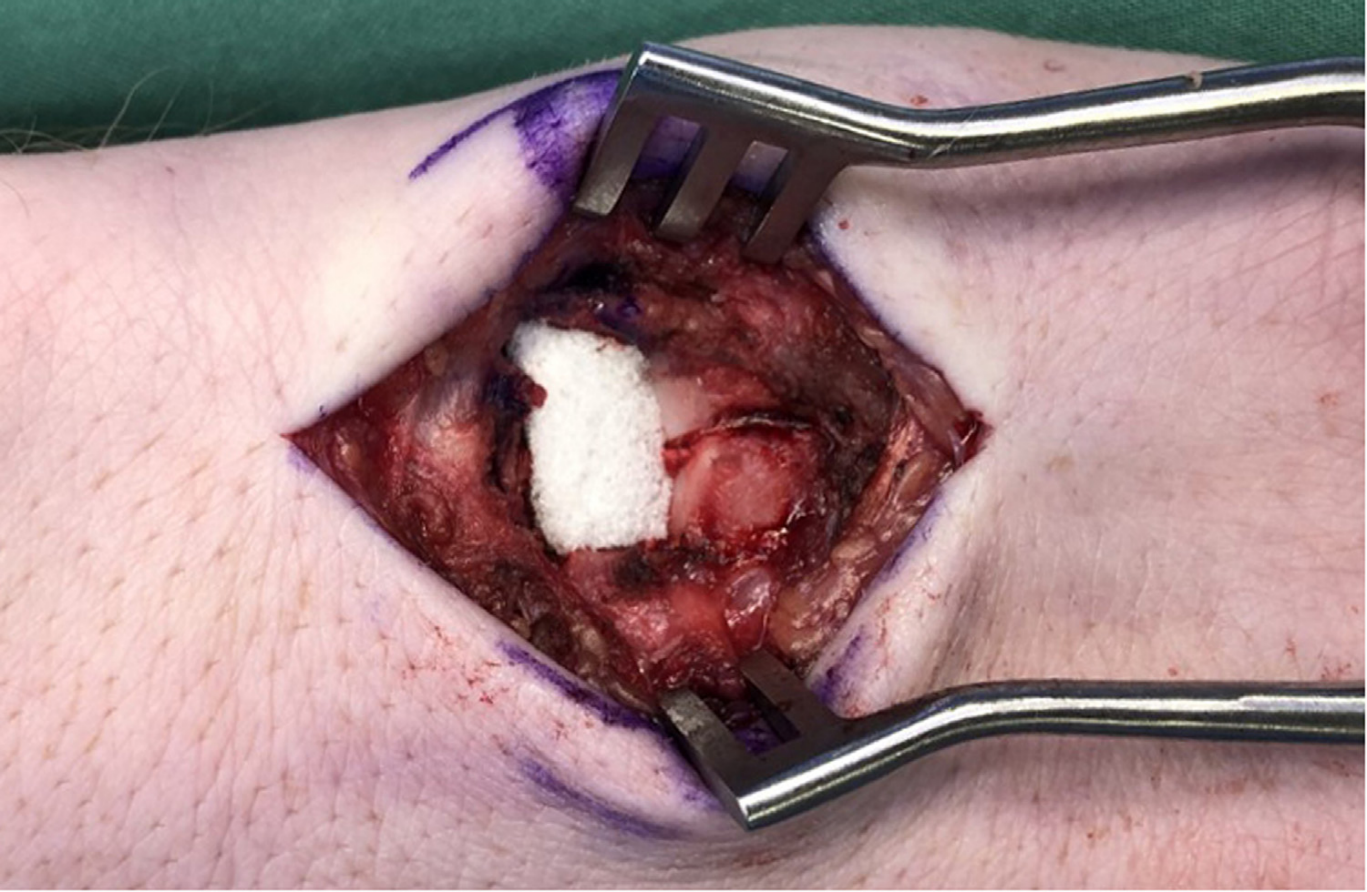
Gel foam.

**Figure 3:**
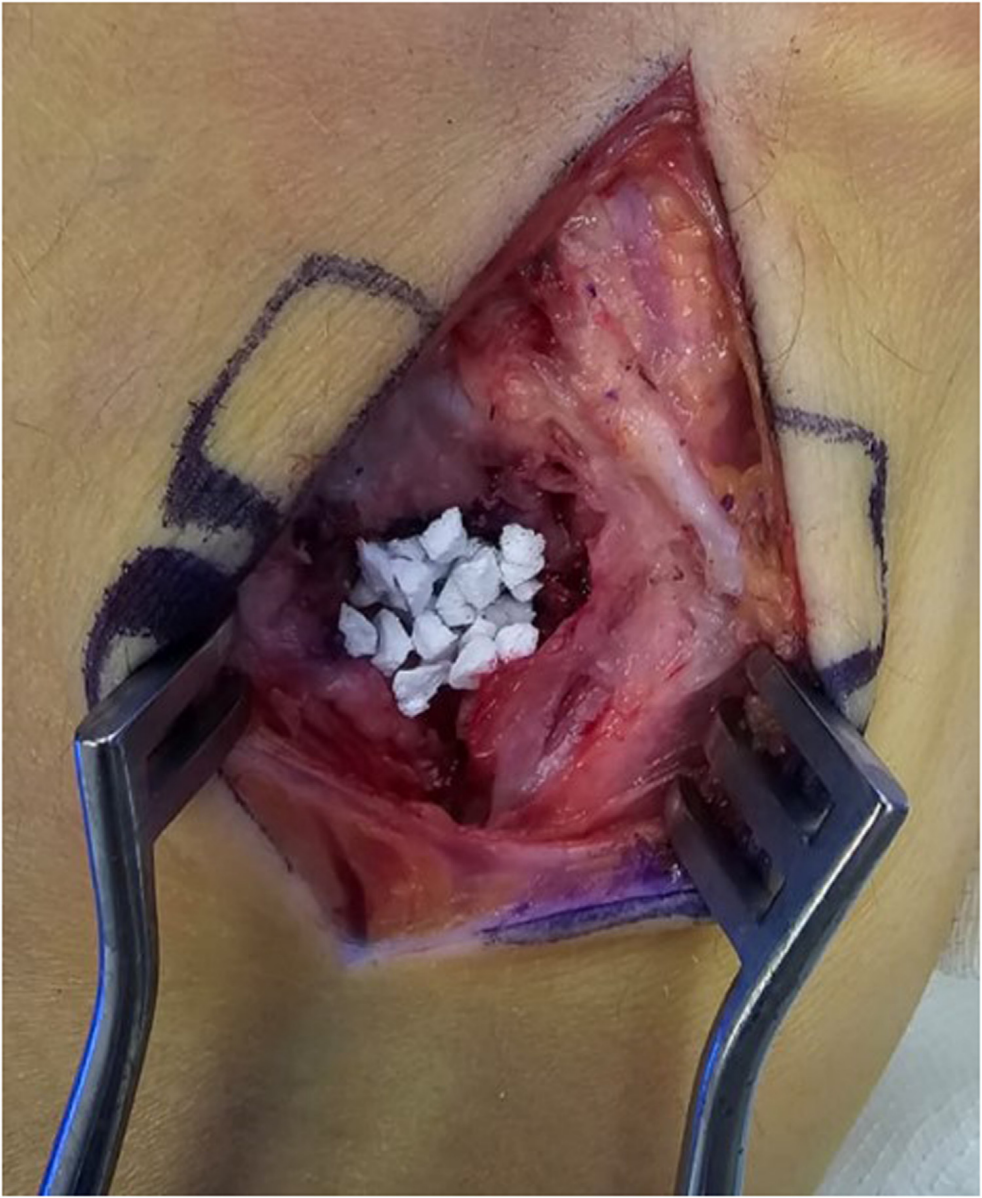
Synthetic bone graft.

**Figure 4:**
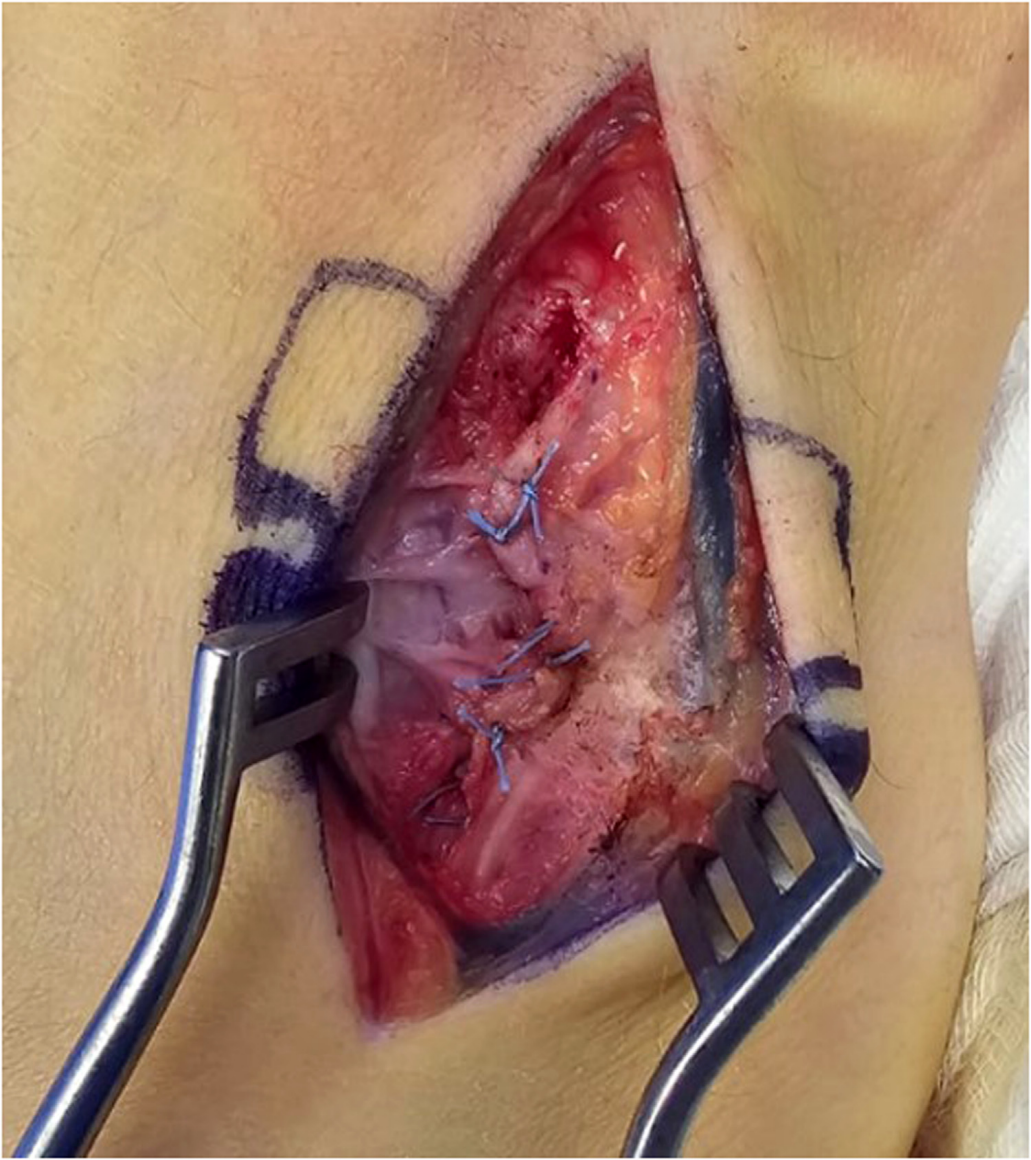
Direct capsule repair.

A total of 14 patients underwent HHA during the study period. Six patients underwent harvest of a vascularized osteochondral hamate graft, while 8 patients received non-vascularized grafts. Hamate defect closure was performed using four techniques Table 1. Nine patients had no reconstruction of the donor site defect Figure 1, one patient had the defect packed with autologous bone graft from the PIP joint fracture site, and one patient had gel foam inserted into the defect. Figure 2. The remaining 3 patients had the defect filled with synthetic bone graft Figure 3. For dorsal capsule closure, 6 patients underwent repair using an ipsilateral extensor retinaculum graft. The other 8 patients had the capsule closed primarily using 3–0 Vicryl sutures Figure 4.

**Table 1:** Reconstructive techniques used at the hemi-hamate donor site.

Hamate defect closure	Patients, n
No Reconstruction	9
Autologous Bone Graft	1
Gel Foam	1
Synthetic Bone Graft	3

At the final 6-month follow-up, all patients demonstrated a full, pain-free ROM in flexion/extension and radial/ulnar deviation as compared with that in the uninjured side. VAS pain scores were 0 both at rest and with activity. Serial radiographs showed maintained joint spaces and carpal alignment without evidence of instability or subluxation. There was no differences were observed in wrist mobility, pain scores, or radiographic outcomes regardless of how the donor site defect was reconstructed or which capsular closure technique was used. Lastly, no postoperative complications were

## Discussion

This study's findings on HHA for the management of comminuted PIPJ fractures reveal significant insights into the efficacy and safety of this surgical intervention, particularly from the perspective of donor site outcomes. Notably, our study documented a complete absence of postoperative complications related to the donor site, with all patients achieving full, pain-free ROM at the 6-month follow-up. This outcome holds irrespective of whether the osteochondral hamate graft was vascularized or non-vascularized, and independent of the technique used for the reconstruction of the donor site defect or the dorsal capsule closure method.

Comminuted, intraarticular PIP joint fractures involving greater than 50% of the middle phalanx base have a high complication rate if treated non-operatively. Surgical stabilization is recommended to restore a stable, congruent joint and allow early motion.^3^^,^^4^^,^^7^, ^8^, ^9^ Multiple papers have been published demonstrating the HHA as a viable stabilization technique, with results up to 8 years of follow-up demonstrating ongoing stability and good patient-reported outcomes using the Quick DASH.^10^, ^11^, ^12^, ^13^, ^14^ However, outcomes can be variable. Postoperative complications including tenderness at the graft site, ulnar collapse, subluxation, and PIP joint contracture have been reported in several case reports and series.^4^^,^^5^^,^^13^, ^14^, ^15^, ^16^, ^17^, ^18^ A primary concern is the biological viability of the hamate graft over time.^19^^,^^20^ When harvested as a non-vascularized graft, intermediate to long-term follow-up has demonstrated joint space narrowing, graft subsidence, and the early development of osteoarthritis in up to 50% of patients.^3^^,^^10^ Use of a vascularized osteochondral flap including the hamate bone and overlying soft tissues has been proposed to improve graft survival,^5^ but long-term studies are lacking.

While outcomes following PIP joint reconstruction have been evaluated, less is known regarding donor site morbidity with HHA, especially when performed as a vascularized free flap. Documented problems include persistent tenderness,^13^^,^^21^ subjective weakness, and neuropathic pain from nerve tethering in scar tissue^4^ reported in one case. Donor site pain is one of the most common postoperative complications, reported in up to 30.1% of patients.^12^^,^^22^ However, reports are variable and objective data are limited, with one systematic review finding donor site pain or instability to be present in only 3% of cases (4 of 133 patients).^23^

Only two case reports specifically discuss the hamate donor site reconstruction. Frueh et al. described capsular repair with absorbable sutures in one patient.^19^ Afendras described packing the defect with local soft tissue without bony or capsular repair.^10^ No studies have systematically analyzed donor site closure techniques or standardized rehabilitation protocols.

This case series aimed to assess wrist function, pain levels, and carpal alignment following HHA using different reconstruction methods. We found no limitations in ROM or increases in pain scores regardless of whether the hamate defect was left open, packed with bone graft or substitute, or directly closed with capsule repair. Maintenance of normal carpal alignment was also universally achieved.

The size of hamate bone graft required to reconstruct the PIP joint is dependent on the size of the defect caused by the injury. Cadaveric and morphological studies on the index, middle and ring finger phalangeal bases suggest that they are not reliably distinguishable from one another, with an average surface area of 0.85 +/- 0.04cm^2^.^24^ The little finger phalangeal base, however, is consistently smaller, with an average surface area of 0.59 +/- 0.04cm^2^. Further studies have found significant variation in hamate morphology,^25^ whereas phalangeal base surface area tends to be more uniform.^24^ These variations make it difficult to predict the size of the hamate defect preoperatively, and whether bone substitute may be required for donor reconstruction.

Based on our results, the hamate defect size after HHA is likely small enough that formal reconstruction may not be necessary if the dorsal capsule is competently repaired. This prevents migration of the fourth and fifth CMCs. Joint stability is further maintained by the palmar, radial and ulnar ligaments, which are all left intact in the procedure.^19^ Further biomechanical studies are needed to clarify the role of bone grafting versus soft tissue closure alone.

This study provides some of the earliest evidence to support that the safe return to unrestricted wrist motion and function is achievable after HHA irrespective of the donor site closure method. We implemented a standardized postoperative splinting and gradual mobilization protocol based on known phases of bone and soft tissue healing. Strict splinting for 4 weeks prevents early wrist motion and may reduce pain during capsular healing. Thereafter, gentle progressive ROM allows return to activity without excess stress. Further investigation is required to validate optimal rehabilitation guidelines.

There are limitations to this study, primarily related to the small sample size and retrospective design. Only 14 patients were included, which underpowers comparisons between the different reconstruction techniques. Variations in surgical decision making and rehabilitation could also influence the results. Nevertheless, this represents the largest case series to date evaluating vascularized HHA donor site management and morbidity. Our outcomes indicate that major postoperative wrist dysfunction is avoidable, which contributes useful clinical knowledge regarding this technique.

In this retrospective case series involving 14 patients with comminuted intraarticular PIP joint fractures, the HHA and free-HHA emerged as an effective method for restoring stability and congruity, underscoring multiple viable options for hamate donor site reconstruction. This study highlights the importance of soft tissue closure, particularly dorsal capsular repair via direct capsular repair or extensor retinaculum grafting, in preventing postoperative complications and maintaining ligamentous integrity, without significantly impacting wrist function, pain, or carpal stability at 6-month follow-up. Given the absence of high-quality comparative evidence, the choice of surgical technique for hamate donor defect repair, which appears equally crucial as bony reconstruction, often hinges on surgeon preference and experience. Prospective comparative trials are recommended to ascertain the optimal strategy, particularly considering the satisfactory functional outcomes demonstrated by these methods.

## Ethical Approval Statement

Institutional review board approval was obtained prior to commencing the study.

## Declaration of competing Interest

The authors declare no conflict of interests.
